# Recent Trends in Valorization of Food Industry Waste and By-Products: Encapsulation and In Vitro Release of Bioactive Compounds

**DOI:** 10.3390/foods12203823

**Published:** 2023-10-18

**Authors:** Mriganka Shekhar Borah, Ajita Tiwari, Kandi Sridhar, Kairam Narsaiah, Prakash Kumar Nayak, Baskaran Stephen Inbaraj

**Affiliations:** 1 Department of Agricultural Engineering, Assam University, Silchar 788011, India; 2 Department of Food Technology, Karpagam Academy of Higher Education (Deemed to be University), Coimbatore 641021, India; 3 Agriculture Engineering Division, Indian Council of Agricultural Research, New Delhi 110012, India; 4 Department of Food Engineering and Technology, Central Institute of Technology Kokrajhar, Kokrajhar 783370, India; 5 Department of Food Science, Fu Jen Catholic University, New Taipei City 242062, Taiwan

**Keywords:** food waste, valorisation, waste management, encapsulation, release behaviour

## Abstract

Food by-products and waste are a boundless source of bioactives, nutraceuticals, and naturally occurring substances that are good for human health. In fact, a lot of by-products and wastes are generated by several food businesses. Therefore, waste management and by-product utilization are the most important aspects of the food sector. According to various studies, many bioactive compounds such as phenolics, carotenoids, and proteins can be recovered as feed stock from various industries’ by-products and wastes using potential technologies. As a result, current trends are shifting attention to the sustainable valorisation of food sector waste management and by-products utilization. Thus, the circular economy principles have been applied to the field of food science. The aim of the circular economy is to ensure environmental protection and promote economic development while minimizing the environmental impact of food production. All of these aspects of the circular economy, at present, have become a challenging area of research for by-product valorisation as well. Hence, this review aims to highlight the emerging trends in the efficient utilization of food industry waste and by-products by focusing on innovative encapsulation techniques and controlled release mechanisms of bioactive compounds extracted from food industry waste and by-products. This review also aims to suggest future research directions, and addresses regulatory and toxicity considerations, by fostering knowledge dissemination and encouraging eco-friendly approaches within the food industry. This review reveals the role of encapsulation strategies for the effective utilization of bioactive compounds extracted from food industry waste and by-products. However, further research is needed to address regulatory and toxicity considerations of encapsulated bioactive compounds and health-related concerns.

## 1. Introduction

Around the world, the food sectors, yearly, produce a significant amount of food waste and by-products. In the course of processing food, 38% of food waste is produced [1]. Food wastes come from a variety of sources, such as by-products from bred animals like carcasses, hides, hoofs, heads, feathers, manure, offal, viscera, bones, fat and meat trimmings, and blood; wastes from seafood such as skins, bones, oils, and blood; waste from the dairy processing industry such as whey, curd, and milk sludge from the separation process; and peelings, stems, and seeds from vegetables. Because of their low biological stability, large nutritional value, high concentration of organic components, high water activity, poor oxidative stability, and optimal enzymatic activity, these food industry wastes should not be disposed of in the environment. The enormous quantity of food waste and its microbial decomposition may have a negative impact on the environment, human health, and waste treatment costs, adding to the cost to the food producer. Effectively utilizing by-products has a positive impact on both environmental pollution and the nation’s economy. The development of the food industries depends critically on effective waste management and by-product utilization. However, because of their advantageous nutritional and rheological characteristics (such as polysaccharides, proteins, lipids, and fibres, as well as flavouring agents, phytochemicals, and bioactive substances), food waste streams offer a promising source of useful chemicals that may be used. Nowadays, consumers seek natural, organic, or healthy food that is safe. Because of consumers’ growing interest in functional foods, the demand for natural foods has increased. For functional foods globally, about $100 billion in demand was predicted in 2013 [1]. The creation of functional foods requires the integration of particular health-beneficial ingredients. Cancer, diabetes, and cardiovascular illnesses are the main causes of threat to life worldwide. The use of functional foods in the diet could prevent certain disorders. 

Microencapsulation and encapsulation technology, defined as the technology of packaging solid, liquid, and gaseous components in a continuous film as a coating to produce capsules from the micrometre to millimetre range in size, may be a valuable and specialized alternative to maintain bioactive chemicals [1]. Due to its useful properties, microencapsulation is widely used by the pharmaceutical and food industries. The functional qualities of microencapsulates encourage easier handling and transportation of bioactive substances and minimize or prevent the release of the core material until encountered, as well as decrease any oxidation reaction of the core material when in contact with environmental factors such as heat, moisture, air, and light [2]. Therefore, this review provides a comprehensive overview on emerging trends in the effective utilization of food industry waste and by-products by emphasizing innovative encapsulation techniques and controlled release mechanisms employed for bioactive compounds that are extracted from food industry waste and by-products. Furthermore, this review aims to assess the sustainability implications, explore applications within the food industry, and the challenges encountered. 

## 2. Microencapsulation Applications in Food Industry: Global Trends 

According to the trends in the food industry mentioned above, global issues such as climate change, population ageing, food waste, unfair trade, and easier access to information are changing consumer attitudes towards purchasing healthy, plant-based, sustainable, and socially conscious food. The home and personal care industries’ growing need for microencapsulated perfumes, bleach activators, and anti-bacterial chemicals is anticipated to accelerate market expansion. The controlled and continuous release of pharmaceuticals using technology is projected to be one of the main drivers of industrial growth in the pharmaceutical sector.

The microencapsulation process provides flavours and fragrances used in food and beverage items with an effective texture mixing, enticing aroma release, and a tasty flavour. Food flavours can be microencapsulated to prevent evaporation, oxidation, and heat degradation as well as to prolong shelf life. This property of microencapsulates is expected to increase the demand for microencapsulated flavours in different food applications.

The COVID-19 epidemic had a negative impact on many businesses. However, the microencapsulation sector experienced sharp growth as a result of widespread demand. Currently, microcapsules are found in a variety of products, including dairy, meat, poultry, and baked goods. Microencapsulation has also been used in the pharmaceutical business to enhance medication-release characteristics, cover bitter taste, boost stability, and enable individualized drug administration.

**Figure 1:**
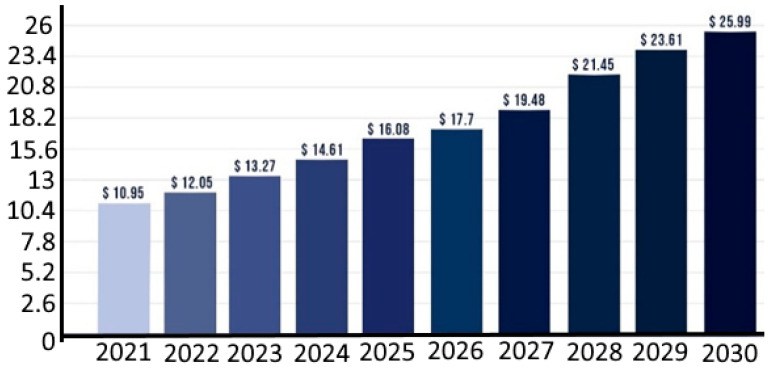
Microencapsulation market size from 2021 to 2030 (USD billion). Source: VisionResearchReporrt (https://www.visionresearchreports.com/) (accessed on 2 September 2023) [11].

In fact, there is an increased interest in the global encapsulation market size (Figure 1). Additionally, as the population ages, health care is receiving more focus [3]. As a result of this, the food and pharmaceutical sectors have started to view functional foods as a promising business with enormous growth potential. In fact, since 2005 [4], the number of patents and scientific research articles relating to functional meals has increased exponentially. In addition to providing macronutrients, vitamins, and minerals, functional foods may also contain active substances such as antioxidants, prebiotics, probiotics, enzymes, and phytonutrients which provide additional health benefits. Their perception as healthy is upheld by the presence of these bioactive substances that promote health in a dietary matrix. Additionally, such functional food has a favourable effect on the final cost of the product.

Microencapsulation, in general, is a dependable technology to address problems associated with the preparation of functional food and to offer additional benefits like controlled or targeted release, increased stability, dissolution, bioavailability, and low ability. 

More individuals are adopting an active lifestyle and realizing the benefits of sports nutrition products, which is causing this nutrition-rich category of products to grow significantly [3]. As these products gain popularity, portable and practical formats (such as drinks, snacks, gels, chews, bars, and shots) are being redesigned. Microencapsulated elements are included in redesigned products to enhance their performance. Therefore, microencapsulation is required to address not just stability but also organoleptic concerns, which are crucial to such products. The “fat is back” movement has led to an increase in the inclusion of specific fats into food matrixes, such as polyunsaturated omega-3 fatty acids [4]. These specific fats need to be concealed since they are easily oxidised and have an unpleasant odour and taste [5]. Additionally, food additives are being reduced or eliminated in accordance with the clean label and healthy living trends. In this situation, the flavour dose can be decreased while maintaining its perception through flavour boosting via microencapsulation [5]. Additionally, it has a favourable effect on the final cost of the product.

A broad variety of conceivable blends could be made possible by microencapsulation since it has the potential to effectively protect and isolate each constituent [5,6]. While research has primarily concentrated on preventing interactions, enhancing the stability of bioactives, and minimising undesirable side effects, there is a growing interest in creating tailored-delivery microcapsules [7,8]. Because customers are aware of the benefits of methods like microencapsulation [9], it will be essential to leverage technical solutions to set products apart from those of rivals [10]. In conclusion, the microencapsulation of food ingredients has evolved into an outstanding instrument for meeting the needs and requirements of consumers as global food consumption habits change. The global microencapsulation market size was estimated at around USD 10.95 billion in 2021 and it is projected to reach around USD 25.99 billion by 2023 [11].

## 3. Relevance of by-Products to Food Industry

The orange pomace’s high fibre content made it ideal for products that needed better water/oil holding and binding properties, such as those that needed a high-water hydration capacity (4.40 mL/g) [12]. Its rich nutritional makeup includes a large amount of dietary fibre (40.47%), less fat (2.14%), and a lot of minerals [13]. Apple pomace has exhibited excellent viscoelastic qualities that could enhance product-structures. Currently, functional foods and food additives are manufactured with pectin and fibre derived from apple pomace. Pectin is also used as an excipient and drug carrier in the pharmaceutical industry. In the Asian market, polyphenolic extracts are also offered as nutritional supplements and food additives [12]. The enormous health benefits of apple peels and apple polyphenols, including their antioxidant, antihypertensive, anti-cancer, anti-diabetic, and hypolipidemic qualities, may create new opportunities for their industrial usage. Xoconostle fruit cultivars, as potential sources of antioxidants for foods, medications, and by-products of the colourant industry, have revealed a high concentration of glucose, citric and linoleic acids, tocopherols, and isorhamnetin-O-(di-deoxyhexosyl-hexoside), as well as relevant antioxidant properties [12]. By-products could be utilised as beneficial food additives rather than being discarded, particularly for formulations that are high in antioxidants. Xoconostle (acidic cactus pear) and its by-products have recently attracted a lot of attention from consumers and medical professionals due to the discovery of their health-promoting potential applications, such as antihyperglycemic, antihyperlipidemic, hypocholesterolaemia, anti-inflammatory, antidiuretic, antiulcerogenic, and immune-stimulating activity, as well as the prevention of some cancers [13]. The amounts of polyphenols, ascorbic acid, and tocopherols in the mesocarp of this xoconostle fruit have been measured by researchers. The pericarp of the xoconostle fruit has the highest anti-oxidant activity (62.96%), followed by the mesocarp (42.27%), and the endocarp (51.70%) [12]. Pomegranate peels and seeds, a by-product of the pomegranate juice and concentrate industries, have a number of therapeutic and nutraceutical uses. A waste and by-product of processing apple fruit is called apple pomace. Apple pomace has a larger proportion of total dietary fibre (74%), along with useful properties like density, the capacity to store both water and oil and to swell, and its glucose dialysis retardation index of 36.91% [12]. The majority (roughly 95%) of the generated biomass is made up of skin or pulp tissues, which are made up of cell wall polysaccharides (such as pectin, cellulose, hemicellulose, and gums) and phenolic compounds attached to the skin, such as dihydrochalcones, flavonols, flavanols, and phenolic acids. Apple pomace is a crucial raw material for the extraction of pectin [13]. The most prevalent phenolic component in apple pomace extracts, phlorizin, serves as the basis for a novel class of oral anti-diabetic medications. Inhibiting sodium-glucose co-transporter-2 (SGLT 2) can treat type 2 diabetes. A recently patented method allows for the enrichment and purification of dihydrochalcones from unwanted ortho-dihydroxyphenol molecules that are covalently bound to proteins and susceptible to oxidation. While contagious polyphenoloxidases are used to produce colours from apple pomace, a novel enzyme-assisted method can be used to extract anthocyanin-based pigments from grape skins without the need for sulfite [12]. As a result, anthocyanins and phlorizin oxidation products are excellent alternatives to synthetic azo colours, some of which have been linked to health hazards. As an alternative to soy and egg protein that is free of toxins and low in antinutrients, de-oiled sunflower press cake is a promising source of nutritive protein [13].

Poly-phenolics are the primary antioxidants found in star fruit (*Averrhoa carambola* L.), which is a good source of natural antioxidants. It was discovered that star fruit residue, which is often discarded during the preparation of juice drinks, has substantially higher antioxidant activity than the juice that was extracted [14]. Strong antioxidant activity in the residue extract delays the oxidative rancidity of soya bean oil. When included in functional food products, residue powder may provide health advantages due to its high ingredient content and potent antioxidant activity. Dietary sources of natural antioxidants for the prevention of degenerative illnesses include fruits and vegetables. The investigation of common sources of cancer prevention agents and their potential for nutraceutical and practical foods has been prompted by the inescapably growing market for these foods. Because of its phytochemical content, apple peel has been considered a high-quality dietary component for foods that promote good health [14]. Pectins are present in the majority of natural product pomaces and, following extraction and filtering, can be used as gelling agents in a variety of food products, including jams, fillings, desserts, and so forth. Pomace can also offer additional ingredients to other foods, such as dietary fibres, lactic acid, colours, vinegar, natural sweeteners, and cellulose [13]. Some organic tropical products (such papain in papaya or bromelain in pineapple) can be used as meat tenderizers, in washing powders, or to ferment lager because they can alter protein structure [14].

Aloe vera, also known as *Aloe barbadensis* M., is an herb with an astringent flavour that is widely utilised in a variety of medical and therapeutic treatments [15]. This plant’s extracted gel has shown excellent antioxidant activity that was on par with synthetic antioxidants like butalated hydroxyl toluene (BHT) [15]. Due to this plant’s high content of polysaccharides and glucosides, it is believed to have antioxidant properties. As a result, aloe vera gel processed for use in food technology may prove to be a valuable source of antioxidant components. Apple peels are a significant by-product of the apple industry, and each year, crores of tonnes of these peels are wasted owing to improper or incomplete processing [15]. Green coconut pulp is used instead of milk, fat, gums, and emulsifier in the formulation of ice cream due to Brazil’s strong rise in green coconut water consumption and waste from the industry [15]. A total of 93.2% of the positive responses, as determined by sensory evaluation, fell within the hedonic scale values of 8 and 9. According to these findings, coconut pulp was used to create foods without lactose, cholesterol, fat, or free milk [16]. Tomato waste polysaccharide, which resembles a xyloglucan biopolymer in structure, was examined for its biological activity and it was revealed that it was anticytotoxic in a Brine Shrimp bioassay [16]. In addition, it has also shown a significant antioxidant activity. The rheological characteristics and biological activities of lemon and Granadilla polysaccharides, which have xylan- and pectin-like structures, respectively, were also examined. Both compounds were confirmed to be anticytotoxic substances [16].

Inulin, fibre, minerals, and beneficial phenolic components are all abundant in globe artichokes. Additionally, preparations from artichoke leaves have long been utilised as a social remedy, particularly for liver complaints. The cynarin (1, 3-O-dicaffeoylquinic acid) component of these extracts has frequently been blamed for the globe artichoke’s medicinal effects. Artichoke leaf extracts have demonstrated hepatoprotective, anti-HIV, anticarcinogenic, antibacterial, bile-expelling, antioxidative, and urinative effects in a variety of pharmacological studies. They have also been shown to be able to suppress cholesterol production and LDL oxidation [17]. By-products from the processing of globe artichokes, such as the leaves, outside bracts, and stems, represent a significant amount of waste that could be used as a source of phenolics and should be viewed as a raw material for the production of dietary supplements and nutraceuticals. Inulin is a member of a class of fructose-based polysaccharides known as fructans. Because humans lack the enzymes needed to hydrolyze fructans, fructans cannot be processed in the small intestine. Inulins have recently attracted attention in the nutraceutical industry because of their significant impact on gut microbiota composition, positive effects on mineral absorption, beneficial effects on blood lipid composition, and potential prevention of colon cancer. In addition, inulin is a low-calorie fibre with potential for application in the creation of foods with less fat. A recently popularized food that is full of healing compounds is the globe artichoke [18].

Onion by-product, which is a source of antioxidant and anti-browning bioactive chemicals, has better qualities that could make it a better culinary ingredient. Due to changes in the organoleptic and visual features of the product, one of the primary problems for the food industry today is to maintain the development of enzymatic cooking before or during the processing of fruits and vegetables [19]. The fact that the phenolic compound content decreases during enzymatic browning means that there will be a quality loss, which is another important consideration. Sulfhydryl (SH or thiol) groups are effective PPO inhibitors. The thiol compounds found in onions are, therefore, thought to be the active ingredients responsible for onion’s PPO inhibiting action. To prepare for the scorching that PPO would cause, onion extracts can be used as a natural food source [20].

## 4. Encapsulation Technologies

**Figure 2:**
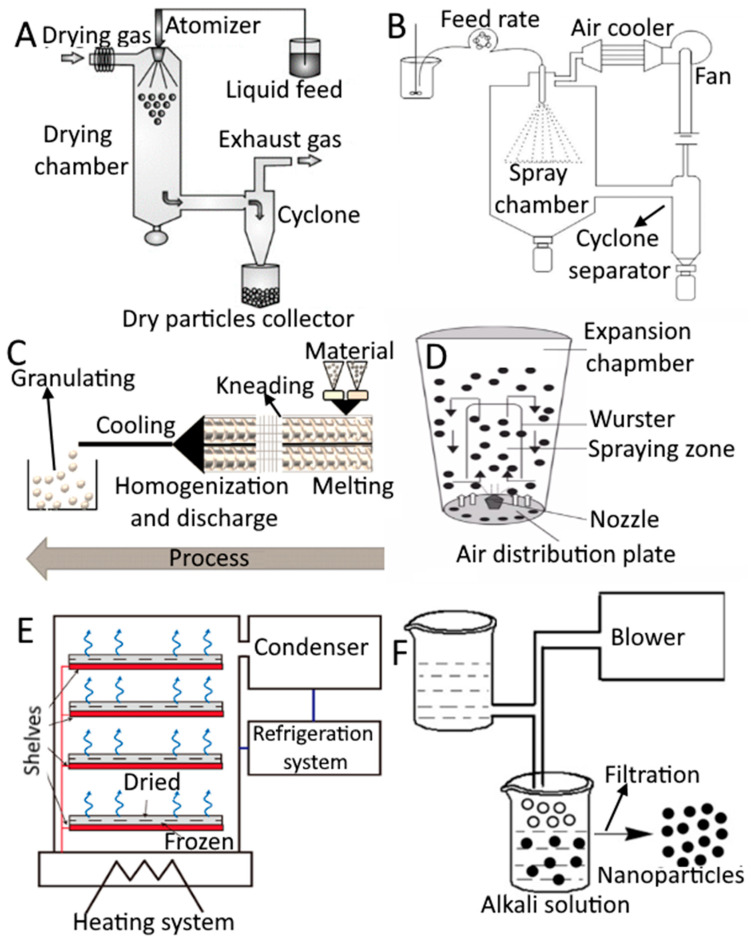
Different types of techniques used for encapsulation. Spray drying (**A**), spray chilling/cooling (**B**), extrusion (**C**), fluidized bed coating (**D**), lyophilization (**E**), and coacervation (**F**). (**A**,**D**,**E**) is adapted with permission (copyright © 2015 Elsevier B.V., Amsterdam, the Netherlands; copyright © Institute of Food Science and Technology, London, UK; copyright © 2021 Elsevier Inc., Amsterdam, the Netherlands) from Sosnik and Seremeta [32], Madene et al. ([33], and Chadha [34], respectively, while (**B**,**C**,**F**) is adapted from Okuro et al. [35], Priyanka et al. [36], and Vilas et al. [37], respectively, and is an open access article (copyright © 2013 Faculty of Food Technology and Biotechnology, University of Zagreb, Zagreb, Croatia; copyright © 2019 by authors; copyright © 2020 by authors) distributed under the terms and conditions of the Creative Commons Attribution (CC BY) license.

There are numerous ways to encapsulate food components in coating materials. The physical and chemical characteristics of the core and coating materials as well as the intended use of food ingredients determine the choice of the microencapsulation technique. In Table 1 and Figure 2 the numerous techniques used to prepare microencapsulated food systems and the microencapsulation technologies that are used to encapsulate food ingredients are shown. Microencapsulation is currently a viable method for achieving a very wide range of capabilities because of the development of sophisticated shell materials and technologies. Any type of trigger, including pH change (enteric and anti-enteric coating), mechanical stress, temperature, enzymatic activity, time, osmotic force, etc., can be employed to cause the release of the encapsulated substance. However, compared to the pharmaceutical or cosmetic industries, cost considerations in the food industry are far stricter. In general, three precautions must be taken when creating microcapsules: creating a wall around the material, making sure there is no leakage, and making sure unwanted things are kept out. Extrusion coating, fluidized-bed coating, liposomal trapping, lyophilization, coacervation, centrifugal suspension separation, co-crystallization, and inclusion complexation are some encapsulation processes (Table 1) [21]. The choice of coating materials and the microencapsulation technique are interrelated. The appropriate method or coating material is chosen based on the coating substance or application method used. Depending on the substance to be coated and the qualities needed in the finished microcapsules, coating materials, which are essentially film-forming materials, can be chosen from a large variety of natural or synthetic polymers. The functional qualities of the microcapsule and how it might be employed to enhance the performance of a certain ingredient are mostly determined by the composition of the covering material [22]. The following qualities should be present in a perfect coating material.

- Good rheological characteristics at high concentration and straightforward workability during encapsulation;
- The capacity to stabilise the created emulsion and disseminate or emulsify the active substance;
- Non-reactivity during processing and after extended storage with the material to be enclosed;
- The capacity to contain the active substance inside its structure while being processed or stored;
- Under drying or other de-solventization conditions, the ability to entirely release the solvent or other ingredients utilised during the encapsulation process;
- The capacity to offer the active material the best defence possible against environmental factors (such as oxygen, heat, light, and humidity);
- The food sector accepts solvent solubility (such as ethanol and water);
- The active core materials’ chemical inertness;
- Affordable and food-grade status.

### 4.1. Spray Drying

The most popular technique for microencapsulation is spray-based microencapsulation. Various microencapsulation methods based on spraying or atomization exist, and aerosol generation, which involves the suspension of tiny drops, enables the gas phase [38]. The oldest and most-used method of microencapsulation in the food business is spray drying. An aerosol is created in this technique by atomizing a mixture of shell and core materials that have been dissolved, dispersed, or emulsified in a solvent. The solvent is subsequently vaporized by heat, forming tiny solidified droplets (microspheres) that enclose the interest-containing molecules. However, there are additional atomization techniques that depend on the shell material freezing or gelling, such as spray chilling and spray freezing. These involve the freezing or thermal gelation of the shell as a result of the atomization of a mixture of molten core and shell material in a cold chamber. Consequently, the droplets harden to create microspheres when they come into touch with the cool air [26,27].

Some benefits of this approach include its adaptability to various encapsulating agents, affordability, flexibility, ability to be scaled up simply, and use with a wide range of materials. The successful use of this approach in encapsulation has been demonstrated in numerous studies. In Cardamom, spray drying was used to encapsulate the oleoresin inside a mixture of modified starch, gum Arabic, and maltodextrin, and the findings revealed enhanced oleoresin protection [27]. Spray drying was used to successfully encapsulate the flavour of sumac in sodium chloride [28]

Despite the fact that spray drying is one of the most widely used methods for microencapsulation and that it has several purported benefits as well, certain research has highlighted some of the method’s disadvantages. When hot air is used to dry omega-3 fatty acids, the dried powder has particles with a highly porous structure, making the powder more susceptible to oxidation and shortening the powder’s shelf life [26]. A similar outcome was observed while creating spray-dried fish oil powder. As a result, it may be claimed that the same process may work well for encasing one type of material but may not work well for another [28].

### 4.2. Spray Chilling/Cooling

In contrast to spray drying, which uses hot air, spray chilling uses cooled or chilled air to atomize the material to be encapsulated with the carrier. In the case of spray cooling (45 to 122 °C), the exterior material is often vegetable oil, and in the event of spray chilling (32 to 42 °C), it is a hydrogenated or fractionated vegetable oil [26]. The drawback of the latter method is that it might need special handling and storage conditions. For ferrous sulphate, vitamin, mineral, or acidulent encapsulation, spray cooling is typically utilised. This technique can be used to encapsulate frozen liquids, heat-sensitive compounds, and substances that are not soluble in common solvents. As the wall material melts, these materials are then liberated. Dry soup mixes, foods with a high fat content, and bakery goods are among the things that can be chilled with a sprayer [26].

Additionally, there is enormous potential for scaling up this method. The microencapsulation of tocopherols within lipid matrix, with an encapsulation efficiency as high as 90%, and the encapsulation of iron, iodine, and vitamin A within hydrogenated palm oil to fortify salt, wherein the microcapsules formed were highly stable, are some successful applications of this technique in encapsulation [28,29].

### 4.3. Extrusion

The research on extrusion started after its initial invention in 1957 [28]. In this period, citrus oils were mixed with glycerine and corn syrup solids at a temperature of 125 °C while being heated by steam [28]. The mixture was then put into a chamber under nitrogen pressure and extruded into a dehydrating liquid like isopropyl alcohol. The material was subsequently divided into smaller (1 mm) pieces and vacuum dried. Later, it was discovered that the dextrose equivalent of corn syrup, the emulsifier and flavour oil level, and the emulsification pressure all helped to improve the quality of the micro-capsules. The benefit of extrusion is that any core is completely rinsed from the outside and that the material is completely separated by the wall material. It is mostly used to extend the shelf life by at least two years and provide visible flavour pieces, vitamin C, and colours [28]. Due to the encapsulated ingredients’ solubility in hot or cold water, applications for dry food include drinks, cakes, cocktails, and gelatin dessert mixes. This technique has also been used to encapsulate numerous flavours [28]. The encapsulates created using this method are often greater in size than those created using any other method, and this technology can be applied to walls made of a restricted number of materials [28].

### 4.4. Fluidized Bed Coating

For fluidized bed coating, the coating material is atomized in a chamber of high-velocity air that is temperature- and humidity-controlled [29,39]. For the best results, particles between 50 and 500 microns in size should be used [39]. The distribution of particle sizes should likewise be limited. The length of time that the particles spend in the chamber determines how much material coats them. This method can be used with solvent-based coatings such as starches, gums, and maltodextrins, as well as hot-melt coatings like stearines, fatty acids, emulsifiers, and waxes. Cool air is utilised to harden the carrier in hot melts, whereas hot air is used to evaporate the solvent in coatings, based on the solvents used [29]. While water-soluble coatings release their contents when water is added, hot-melting components release their contents by raising the temperature or causing physical breaking [29]. Iron can be separated from ascorbic acid in multivitamins and tiny tablets, including children’s vitamins via fluidized bed encapsulation. Many fortified foods, nutritional blends, and dry mixes have constituents that have been fluorescently bed-encapsulated. Salt added to pretzels and meats, lactic acid, sorbic acid, vitamin C, sodium bicarbonate in baked goods, and citric, lactic, and sorbic acids are all encapsulated [29,40].

### 4.5. Liposome Entrapment

Liposomes are one kind of capsule that has more adaptable properties and less fragility than those formed of fat [30]. They have been employed to transport vitamins, enzymes, hormones, and vaccinations to the body. They include one or more lipid layers, making them non-toxic and suitable for food. Size and lipid content differences can alter the permeability, stability, surface activity, and affinity of food. They are simple to manufacture, have a diameter that can range from 25 nm to several microns, and can be freeze-dried for storage. A method to encapsulate them with high efficiency that is simple to scale up and uses moderate conditions suitable for enzymes was developed [30].

The outer layer or layers of liposomes are composed of phospholipids. The hydrophobic groups of the lipids associate with the hydrophobic groups of other lipid molecules, while the hydrophilic portion of the lipids is directed towards the aqueous phase. Because the lipid sheet does not interact with water when it is folded into an aspherical shape, the resulting capsule is exceedingly stable. These membranes trap either aqueous or lipid-soluble molecules, but not both. This method is mostly used to encapsulate flavouring ingredients. From a few nanometres to a micrometre is the size range for liposomes. They were created initially for medical applications [24], and later on, for cosmetic ones [25]. A description was provided of how liposomes were used in the production of cheese in [30].

Phosphatidyl choline, the most prevalent phospholipid in lectins, cannot be economically extracted from soy or egg yolk and is insoluble in water. Whether a single layer or numerous layers are created depends on the phospholipids’ makeup and the method employed [31]. Liposomes are also made of fatty acids, and the source determines how saturated they are. Saturated fatty acids are more abundant in animal sources. They have an impact on the temperature at which a gel changes into a more leaking-prone liquid.

There are three distinct processes used to create liposomes. The lipid formulation is combined with a 2:1 chloroform:methanol solvent solution. A reduced solvent volume is followed by re-dispersion of the lipid/solvent film in an aqueous phase. In this step, liposomes are created using a variety of techniques, such as physical, two-phase, and detergent solubilization. After that, the liposomes are extracted from the water [41].

Over time, the phospholipids in the liposomes either oxidise or hydrolyse. Using freshly prepared lipid and solvents to create the liposomes, limiting excessive temperatures, avoiding oxygen exposure as much as possible, adding antioxidants and metal chelators to prevent charge neutralization by metals, and using appropriate storage conditions can all help to ensure the maximum stability of the product. By utilizing pure solvents and removing as much water as possible, hydrolysis can be reduced to a minimum. To prevent annealing or fusing, the temperature should be kept above the phase transition point. Liposomes under 40 nm are more likely to merge than those over 40 nm [42]. Vander Waals forces will still cause neutral liposomes to cluster; however, adding 5% phosphatidic acid or phosphatidyl glycerol can lessen this [31].

### 4.6. Coacervation

The coacervation method for carbonless paper was patented by the National Cash Register Company in the 1950s [41]. Particle sizes ranging from a few sub-microns to a centimetre were acquired. The carrier has just recently been made of food-grade materials. Despite being effective, this procedure is pricey. It involves dissolving a protein that gels, and then emulsifying another material, such as flavouring oil, into the protein. The item to be coated with the coating is withdrawn from a polymer solution in liquid form, coated, hardened, and collected by centrifugation or filtration. Spray drying or fluidized bed drying can be used to achieve drying. When creating the micro encapsules, the variables of pH, temperature, and content are all significant.

Gelatin is one example of a colloidal solute that can cause coacervation. Coacervation can also be complex when using gelatin and gum acacia [42]. Because each substance has an opposing charge at a low pH, which attracts the other and results in the development of an insoluble complex, gelatin and gum acacia are employed in combination. It is more typical to utilise this viscous solution to coat flavour oil droplets suspended in aqueous media [43]. The wall material becomes harder as the temperature is lowered, but it can be made softer once more using bases, acids, heat, or dilution. In the presence of divalent salts or aldehydes, this reaction is irreversible. Hydrophobic substances like citrus, vegetable oils, or vitamin A can be microencapsulated using hydrophilic coverings like gelatin. It is possible to discharge the contents using hot water, pressure, or a chemical reaction. The core may be water-soluble or immiscible, and the covering may also be hydrophobic [44].

Numerous research studies have been published indicating the effectiveness of this method in microencapsulation. By employing soybean protein isolate (SPI) as the wall material, the coacervation technique was used to encapsulate sweet orange oil [28]. Complex coacervation is the term used when coacervation involves more than one polymer. Polymers, gelatin, and alginate are a few examples of common complicated coacervations. Alginate is solubilized in water separately at a basic pH to obtain negative charges while gelatin is separately solubilized in water at an acidic pH to obtain positive charges. The alginate solution is thoroughly homogenised when the active substance to be encapsulated is added [28]. The gelatin phase and alginate phase are then thoroughly combined, and the temperature is increased until a chemical interaction between the alginate and gelatin begins. A polycationic, polyanionic, insoluble polymer forms all around the active substance, encasing it. Complex coacervation was utilised to encapsulate and stabilise flaxseed oil [28]. The usage of this approach is constrained, though, as it only performs well within a narrow pH range and with specific electrolyte and colloidal solutions [45].

There has been a lot of interest in the use of complicated coacervates made from biopolymers in food products. Capsules with active ingredients can be added to food products for a variety of reasons, such as changing the texture and colour or exhibiting antibacterial and antioxidant properties. The application of encapsulated compounds in food and the release of agents from coacervates inside a food matrix, however, have received very little research. Because of this, there is a larger demand for research on the method and use of complicated coacervation in the food business [39,40,46].

### 4.7. Centrifugal Suspension Separation

The procedures in the relatively new technology of rotational suspension separation involve mixing the core and wall materials before introducing them to a spinning disc [43]. The core elements eventually emerge from the disc covered in leftover liquid. After being taken off the disc, the capsules are refrigerated or dried. The entire procedure can take from a few seconds to many minutes [44]. In this way, solids, liquids, or suspensions between 30 microns and 2 mm can be enclosed. Coatings can range in thickness from 1 to 200 microns and can be made of meltable materials such as diglycerides, polyethene glycol (PEG), and lipids [47]. This continuous, high-speed technique may coat particles, making it ideal for use in food processing. One use is to safeguard foods like aspartame, vitamins, or methionine that are sensitive to or easily absorb moisture [47].

### 4.8. Inclusion Complexation

Beta-cyclodextrin is employed in inclusion complexation because it has seven linked glucose units that are hydrophilic on the outside and hydrophobic in the centre. Less polar molecules take the place of water molecules in the middle of the cyclodextrin [48]. Following that, the complex precipitates out of the solution [48]. The suspension medium can only be water. Using traditional techniques, the precipitate is collected and dried. The cyclodextrin can bind at temperatures as high as 200 °C. However, the bound substance can be released due to the moisture content and temperature of the mouth. Cyclodextrin can be used to combine garlic and onion oils to create molecules with less Odor. This technique can also stabilize the fat-soluble vitamins A, E, and K [48].

The inclusion complexation method is typically employed to encapsulate volatile organic compounds (such as vitamins and essential oils); it is helpful to hide flavours and scents while maintaining fragrances. A higher encapsulation efficiency and increased core component stability were produced by this method. Only a select few specific molecules, such as β-cyclodextrin and β-lactoglobulin, may be encapsulated using this technique.

## 5. Applications and Benefits of Encapsulated Products Derived from Food Industry Waste and By-Products

The incorporation of these components into food products is complicated by the presence of bioactive substances in food by-products, such as phenolic compounds, procyanidins, polyphenols, anthocyanins, vitamins, and polyunsaturated fatty acids, which are sensitive to environmental factors. To generate functional foods with enhanced stability, quality, richness in bioactive chemicals, and aggregated value, encapsulating food by-products rather than employing them as encapsulating materials is also being studied. Encapsulation has been a widely utilised method for incorporating food by-products into foods, according to previous research. These by-products can be successfully incorporated into food items since they are high in bioactive ingredients. In this approach, the waste mimics the ecological burden and is reintroduced into the food chain. The bioactive food products that have been produced as functional foods may contain antioxidant, antibacterial, neurotransmitter, anti-diabetic, antifungal, and other qualities. These by-products are included in a variety of food items, including bakery goods like cookies, cakes, muffins, etc., animal products like beef, chicken, meat, sausages, etc., dairy items like cheese, yoghurt, curd, butter, ice cream, and beverages like orange, apple, and carrot juices. Table 2 lists some recent food items that were created using bioactives that were isolated from various fruits and vegetables by-products.

In a nutshell, food products made with fruit and vegetable by-products are high in fibres and bioactives. The dosage of bioactives needed, the matrix in which they are added, their sensory evaluation, and customer acceptability all affect how much fruits and vegetables are put into food products.

## 6. In Vitro Digestibility and Release Profile of Encapsulated Bioactive Compounds

The human gastrointestinal tract continuously and dynamically subjects the ingested food products to a variety of physical, chemical, and physiological processes until the nutrients and bioactive chemicals that can be absorbed by the body are released. A thorough understanding of the primary physicochemical changes that take place in the various sections of the human digestive system, specifically the mouth, stomach, small intestine, and large intestine is very much important. These skills aid in the creation of effective delivery systems that control digestion and, as a result, aid in the release and absorption of bioactive chemicals in desired or predetermined areas of the human GIT [70]. The ingested goods are subjected to mastication and salivation in the mouth, causing the meal to break down into minute particles and create a food bolus [71]. The food products, therefore, address dilution and body temperature. Saliva has a pH of 7, is 99.5% water, 0.3% proteins/enzymes, including amylase, an enzyme that may hydrolyze carbohydrates and electrolytes that contribute to saliva’s low ionic strength, and includes salt, phosphate, potassium, calcium, magnesium, and bicarbonate. Due to the nature of gastric fluid, food products must contend with stomach motility, a pH ≤ 7, a strong ionic dilution, and enzymes (gastric lipase and protease) which break down lipids and proteins, respectively [72]. Following intestinal peristalsis, the partially digested meal components undergo mineralization with bile salts, neutral pH, high ionic strength, and dilution in the small intestine. Proteins, starches, and lipids are hydrolysed by protease, amylase, and pancreatic lipase, respectively. The main absorption site for the released bioactive compounds in this compartment is the duodenum. In the large intestine, where intestinal peristalsis, a pH range of 7 or slightly less, and a strong ionic force are present, the unabsorbed substances come into contact with these conditions. The microbiota becomes involved in the fermentation of carbohydrates and proteins in this situation [70,73]. The pH changes in the aqueous phase in the human gastrointestinal tract cause the surfactants, proteins, and polysaccharides that make up carrier-based systems to fluctuate in electrical charge during digestion. As a result, their composition, structure, and interactions are changed [70]. The fluctuation in the type and concentration of ions produced by the location of the human gastrointestinal tract and/or the type of foods consumed may have an effect on the electrostatic interactions in carrier-based systems through electrostatic screening or binding effects [70]. In the presence of endogenous (like proteins), exogenous (like surfactants), and internally generated (like lipid digestion products) surface-active components, carrier-based systems’ interfacial compositions and characteristics may vary [70]. There are numerous enzymes that can degrade carrier-based system components in the human gastric and intestinal tracts, and these enzymes are active [70]. Variations in temperature during digestion in carrier-based systems can be observed in the physical state, molecular conformation, or the interactions of certain components, which impacts how digestible they are [70], not to mention that the flow/force profile to which carrier-based systems are exposed could lead to the collapse of their structure [70]. Under in vitro gastrointestinal circumstances, the behaviour of delivery devices with bioactive compound-loaded products has been evaluated. To learn more about the availability of intestinal absorption in this work, static and dynamic digestion models are applied. Cell culture research is considered to further investigate intestine absorption and systolic metabolism. The viability of determining internal bioaccessibility from gut flora may be investigated in future research.

Evaluations of the bioaccessibility and bioavailability of bioactive substances are also taken into account using in vivo techniques [74]. Table 3 provides an overview of various simulated techniques. The parts that follow provide a critical assessment of these subjects. Additionally, some research on this topic is discussed regarding the intestinal absorption of released bioactive compounds as well as the digestion of carrier-based delivery methods.

### 6.1. In Vitro Digestion Models

Many in vitro models, ranging from single static systems to multi-compartmental dynamic systems, have been created to imitate the physiological processes which occur throughout each digestion in the gastrointestinal tract, including the step, pH, and enzymes, among others [45,75,76]. They are designed to be straightforward, adaptable, affordable, and repeatable when evaluating the bioaccessibility of target bioactive compounds [73,76]. On the other hand, this approach makes it possible to lessen the technical, ethical, and financial limitations associated with in vivo investigations [70]. There are now 466 papers that deal with “in vitro digestion models,” of which 279 deal with both “foods” and “in vitro digestion models” at the same time. With 138 articles in the last five years (from 2017 to 2021), a significant amount of progress has been achieved on this subject.

#### 6.1.1. Static Models

Nearly 90% of in vitro digestion research has been carried out with static models [75]. These are single static systems that can imitate the stomach and/or intestinal phases, with mono- or multi-compartmental protocols (a series of stirring vessels is used here) being taken into consideration. To reproduce various sections of the gastrointestinal tract, a single set of initial biochemical parameters (i.e., temperature, pH, concentration of enzymes, and bile salts) is established and maintained throughout the process [72,77]. Pepsin hydrolyses homogenized food at a specific pH (1–2), temperature (37 °C), and duration (1–3 h) to replicate the stomach. In turn, pancreatic enzymes (with or without bile) are used to imitate the intestine at the correct pH (6–7) [76]. The behaviour of various delivery systems under various simulated gastrointestinal conditions has been evaluated in multiple studies using in vitro static digestion models. Some of the newest are compiled in Table 2 [78]. The release of curcumin from ternary complexes made of high methoxyl pectin, surfactants (rhamnolipid, tea saponin, or ethyl lauroyl arginate hydrochloride), and pea protein isolate was studied using a simulated gastrointestinal digestion model made up of stomach and intestinal phases [79]. The ternary compound containing rhamnolipid led to the slowest release of curcumin in the simulated gastrointestinal tract. Additionally, this formulation made it possible to guarantee that curcumin’s useful property was rarely harmed during the digestive process. This in vitro digestion investigation provided convincing evidence that a carrier-based delivery strategy for curcumin encapsulation with applications in industrial food products would be more effective [80]. To evaluate the stability of capsules made with two types of gelatin (ac-id-processed (type A) and alkaline-processed (type B) gelatin) for the integration of Thai Rice berry bran extract, a static simulation model with oral, gastric, and intestinal phases was also developed [81]. It was determined that the net surface charge and gelatin particle size have an impact on the stability of this bioactive molecule during in vitro digestion. The application of a generic, standardised in vitro digestion protocol suggested by the COST INFOGEST network has caused further experiments to be conducted which differ from those described. In fact, despite the in vitro static models’ simplicity, low cost, high throughput, and ability to analyse multiple samples [70,73], one of the main issues with this methodology is the wide variations in digestion parameters that are seen among different models and that make it impossible to compare results across research groups. These factors include, among others [72,82], the quantity of the product sample, the pH, the number and type of enzymes, the quantity of bile salts, the rate of stirring, and the length of each stage of digestion. Accordingly, the use of a uniform protocol is typically taken into account in the most recent investigations [83]. To validate their proposal on coating Pickering emulsions (prepared using whey protein nanogel particles and emulsions) with a dextran sulphate layer to lessen these emulsions’ destabilisation under gastric conditions, for instance, one study used the standardised static in vitro gastric digestion (INFOGEST model) [84]. The results obtained support the formation of stable emulsions in gastric medium, which emerge as a promising method for delivering lipophilic substances that have been encapsulated to the intestine. Microfluidization and ultrasound were used to create high oleic palm oil-loaded nanoliposomes, and how these two production techniques would affect the physical stability and intestinal digestibility of these structures was assessed [85]. Here, the COST INFOGEST network’s standardised protocol was put into practise. No statistically significant changes in the stability of the nanoliposomes were seen after oral phase digestion. While the triglycerides that make up the nanoliposomes were destroyed by enzymatic activity in the intestinal phase, the stomach phase favoured the destabilisation of both types of nanoliposomes. Additionally, the study discovered that two ovalbumin nanoparticles loaded with conjugated linoleic acid underwent physicochemical and structural alterations throughout the stomach and intestinal phases, taking into account the conventional in vitro static gastrointestinal digestion model created by the COST INFOGEST network [86]. The acquired results showed that during, gastric-intestinal simulation, conjugated linoleic acid was retained at significant levels. The standardised in vitro digestion methodology was used to examine the resistance of zein- and ethyl cellulose-based, β-carotene-loaded nanoparticles to gastrointestinal conditions. The digesting media revealed that both carrier-based delivery systems were strongly insoluble, making it impossible for the salts and enzymes contained within to break down the nanoparticles [74]. Because of this, even during the gastrointestinal phase, β-carotene re-release was prevented. Zein nanoparticles had the maximum bioaccessibility of encapsulated beta-carotene in the digestive phase (37 1%). In turn, the potential of bovine serum albumin particles coated with chrysin to be employed as supplements to be added to functional foods using the INFOGEST technique (gastric and intestinal phases) was examined [86]. During in vitro static GI digestion, a significant amount of encapsulated chrysin is kept inside the particles, resulting in the release of 14% of this bioactive substance. To test the effect of chitin nanocrystals on the digestion of maize oil-in-water emulsions (stabilised with tween 80) and the consequent bioaccessibility of β-carotene encapsulated into this lipidic-based delivery system, a standardised in vitro digestion regimen was developed [87]. The results obtained demonstrated that micro chitin decreases the bioaccessibility of β-carotene via limiting lipid digestion. Additionally, some disparities between these results and those obtained using a standard static in vitro digestion model (non-INFOGEST) were discovered by the authors [84]. Even though the same conclusions had been reached, it was found that, when the non-INFOGEST approach was used, there was a reduced percentage of total free fatty acids released and carotenoid bioaccessibility (from 68.9 to 99.6% and from 22.7 to 55.2%, respectively) [88]. In actuality, the first three factors—enzyme activity, calcium contractions, and bile salt—were thought to be the most significant for the changes seen. This is one of the primary variances between the two models. The requirement to perform a back-titration after the small intestine phase in order to identify all of the free fatty acids that were released was noted by the authors as a drawback of the INFOGEST model in their tests.

Although static digestion models have been employed frequently to research the digestion of various foods (they were created with this goal in mind), they are oversimplified and do not accurately simulate the most intricate gastrointestinal conditions and processes [73]. In actuality, the bio-chemical environment in the human body is constantly changing, hence it is impossible to simulate this activity using static models as previously mentioned. Additionally, a lot of media is employed, and the continuous stirring method used does not accurately reflect the complexity of peristaltic movements. Additionally, this technique disregards gastric emptying [75]. Therefore, in vitro dynamic digestion models were created to get around the drawbacks of static models.

#### 6.1.2. Dynamic Models

In vitro dynamic models have become popular in recent years as a cutting-edge technique that can offer a better and more sophisticated digestive simulation method. They incorporate the dynamic elements of the digestive process and can be single or multi-compartmental systems. These include the gastrointestinal tract’s shear, mixing, hydration, and peristalsis phenomena [74]. The goal is for dynamic models to be able to reproduce changes in enzyme concentration, viscosity, pH, particle size, and nutrient partitioning, the transit of digested products, and the continuous discharges of digestive fluids seen in the gastrointestinal tract of the human body [72,74]. This methodology allows for a more accurate assessment of the bioaccessibility of certain bioactive chemicals as well as a better approximation of the mechanisms and processes involved in digestion and the structural modifications of food products. Several in vitro dynamic digestion models have been created and published in recent years with various geometries, physical forces, and biochemistries [89]. The dynamic rat stomach–intestine–duodenum system (DRSD), the dynamic human stomach–intestine system (DHIS), the human gastric simulator (HGS), the TNO’s gastrointestinal model (TIM), the gastric digestion simulator (GDS), the in vitro mechanical gastric system (IMGS), the simulator of the human intestinal microbial ecosystem (SHIME), and the dynamic gastric model (DGM) are some of the most significant dynamic models [72]. As a result, further digestion simulation models have been created and presented [73].

## 7. Release Profile

**Figure 3:**
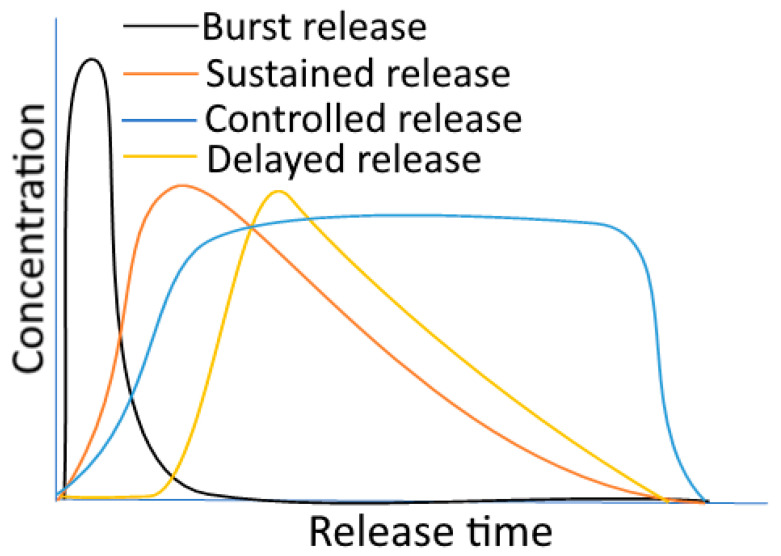
Various release profiles for components in encapsulated food. Figure 3 is adapted with permission (copyright © 2021 Elsevier, Ltd., New York, NY, USA) from Boostani and Jafari [90].

A release profile is defined as the occasions when encapsulated substances are released from a carrier while exhibiting a particular concentration–time curve at the target site. The type of release mechanisms has the biggest impact on release profiles. Figure 3 displays various release profile patterns.

### 7.1. Burst Release

Burst release is the uncontrolled leakage of the encapsulated bioactives during the first few seconds of release, which may also be accompanied by carrier swelling. The amount of discharge in this situation can range from 30 to 60% [91]. Due to this behaviour, a typical release profile—a burst effect (an initial quick release) and a subsequent cumulative profile—can be observed. It may eventually hit a plateau or continue to release energy gradually over time. In terms of both the economic and therapeutic views, the consistent release of the bioactives is preferable, whereas the burst profile is an organized condition of release. For instance, hybrid zein/hordein nanofibers inserted within cellulose nanowhiskers showed a reduced burst effect during the controlled release of the encapsulated riboflavin [92]. Another example involves the incorporation of nisin into chitosan/sodium alginate and Pluronic F68 nanocapsules, which demonstrated an early fast release followed by a prolonged and slow-release behaviour [93]. This burst release may be caused by the presence of nisin on the surface of the carriers. It should be highlighted that a burst effect is acceptable when a compound’s function is improved by its quick release after being encapsulated [94].

### 7.2. Sustained Release

Sustained release is a “long acting” release profile when compared to “rapid” or “conventional” release patterns. In comparison to immediate-release or conventional release profiles, sustained release implies a minimum two-fold reduction in the amount of bioactive that is released [95]. For CR formulations, commonly advised release profiles include delayed and sustained releases. Through the sustained release technique, several medications, like insulin, can have a pro-long release into the bloodstream [94]. In the research on diffusion mechanisms, the release analysis of vanillin from poly-lactic acid (PLA) nanoparticles in one instance revealed an instantaneous burst release followed by a consistent and delayed profile [96]. Additionally, streptomycin was encapsulated in starch nanocrystals with chitosan-based antimicrobial coatings, which showed controlled swelling behaviour and a prolonged release profile [93].

### 7.3. Delayed Release

This technology is made to release the individual components of the food ingredients over time after use [97]. Because of this, in the case of delayed release, the release of the encapsulated component is postponed from a short delay to an exciting moment. Delayed release methods may be able to regulate the release site, like when a bioactive chemical is protected in the stomach before being released into the small intestine or the large intestine (colon-delivery) [94,97]. For instance, nisin contained within alginate/chitosan nanoparticles demonstrated a sustained release with persistent antibacterial efficacy against Listeria monocytogenes [84]. Another instance involved resveratrol liposomes embedded in an alginate gel network, which displayed a delayed release profile with a link between the gel concentration and re-lease rate [72].

### 7.4. Triggered (or Stimuli Responsive) Release

A triggered release profile, also known as a stimuli-responsive release profile, is the sudden release of an encapsulated substance in response to changes in various external stimuli. According to McClements (2014), there are two main categories of triggers: (i) internal triggers like enzymes, pH, glutathione (GSH), reactive oxygen species (ROS), and temperature (within the body); and (ii) external triggers like light, ultrasound, magnetic or electrical fields, and mechanical triggers, which can be useful for active packaging [94]. It has been demonstrated that β-cyclodextrin exhibited a stimuli-sensitive profile when an antimicrobial volatile component was released (as a symptom of insufficient heat processing or storage conditions). High relative humidity in the environment served as a stimulus for such a system [84].

### 7.5. Targeted Release

In this instance, the bioactives’ primary components are released at or close to the intended site of the physiologic activity. Both immediate and extended release properties may be present in targeted release systems [95,97,98]. Passively targeted designs can be used to target bioactive molecules. Active delivery, on the other hand, refers to the process of targeting certain encapsulation systems in pharmaceutical delivery applications by attaching particular ligands (moieties) such as antibodies onto the carriers that bind to their particular receptors, which are antigens on cell surfaces [97]. It has been demonstrated, for instance, that zein nanospheres can be used to direct the release of essential oils (EOs) into the large intestine. Zein nanospheres slowed down small intestine discharge and inhibited gastric digesting. The large intestine, however, showed a quicker release rate [88]. Another illustration of this is small intestine-targeted nano emulsions stabilized by soy protein isolate (SPI) that contain phytosterol and carotene components. Such systems’ contents were released in the small intestine rather than during the gastric digestion [99].

## 8. Safety and Toxicity of Encapsulated Bioactive Compounds from Food Waste

The demand for products that are devoid of synthetic and artificial additives is rising along with the demand for formulations that guarantee the stability and secure delivery of food’s bioactive elements to the intended organs and cells. Numerous food ingredients and nutraceuticals have recently been encapsulated using a variety of technologies [100]. Encapsulation technology is one of the most popular methods for ensuring the stabilisation of delicate components, the controlled release of core material, and the physical separation of reactive or incompatible materials, all of which increase the shelf-life of products [101].

A “nanomaterial” is one that has at least 50% of its total particles with one or more exterior diameters between 1 and 100 nm, according to the European Commission [102]. As a result of processing or cooking, nanomaterials can naturally appear in food structures at a nanoscale scale (for example, in emulsions like mayonnaise), or they can be artificially created [102]. In the final scenario, the substance, such as a nanoemulsion or nutritional nanoencapsulation, can be metabolised or eliminated by the body (for instance, vitamins), or they can endure or be slowly soluble, as with synthetic titanium, nano-silver (an antibacterial agent), and amorphous silicon (an anti-caking agent) (food additive) dioxide [76].

Spray drying, fluid bed drying, extrusion, liposome techniques, centrifugal separation, rotating suspension separation, and electrostatic deposition are some of the methods that could be used for encapsulation [103]. The National Nanotechnology Initiative’s (NNI) key objective is to prevent BCs from deteriorating along with cellular metabolism and digestive processes. This will enable a controlled release of BCs to target tissues affected by biological disturbances [104]. Japan and the European Union, both of which have committed considerable resources to this effort, are other countries that have followed them [105]. Nutraceuticals, gelation and viscosifying agents, nutrient propagation, mineral and vitamin fortification, and the nano-encapsulation of flavours are a few food processing techniques that use nanomaterials [106]. Nanomaterials are currently present in commercially available food; however, there is still a paucity of safety-related knowledge. To warn customers about common food-related products using nanotechnology, the Centre for Food Safety put together a list that includes plastic storage containers, infant bottles, and sweets (M&Ms and Skittles). Laboratory research is still the only place where encapsulation for bioactive chemicals extracted from agro-food wastes is used; a few examples are listed in Table 4.

Following ingestion, nanomaterials may follow one of three biological fates in the gastrointestinal tract: (1) they are completely digested and absorbed; (2) they are partially digested and only slightly release the encapsulated compounds; or (3) they are resistant to digestion and the encapsulated compounds are thrown out of the digestive system or cross the intestine epithelium and enter the bloodstream. The toxicological properties of the ingredients employed in the manufacturing of nanomaterials must be taken into account for the three circumstances, as well as a potential immunological reaction [110,111]. After that, the intestinal epithelium does not reject the nanoparticles [112]. However, as particle size reduces, the bioavailability of the encapsulated BCs would rise, directly affecting absorption and improving health outcomes [113].

The properties of nanoparticles, which are influenced by their compositions and designs, can be used to explain their toxicity [112]. Due to their large surface area, nanoparticles may adsorb digestive enzymes, changing how digestion normally operates. Nanoparticles may concentrate in particular tissues and cause toxicity depending on their composition, size, shape, aggregation state, and interfacial qualities [114]. Reactive oxygen species may be produced in the cells by inorganic nanoparticles [115]. It is possible to modify where bioactive substances are released and absorbed in the gastrointestinal tract, which could have negative consequences on health [113]. Since encapsulation protects the bioactive compounds and increases their bioaccessibility, toxicity may potentially be caused by a greater concentration of these substances [116].

Although there is currently only a small amount of regulation governing the use of nanoparticles in the food business, agencies and governments assert that this legislation guarantees the safety of nano-food products [102]. The European Food Safety Authority (EFSA) created a guidance document in 2011 titled “Guidance for the risk assessment of the application of nanoscience and nanotechnologies in the food and feed chain.” The manufacturing of the nanomaterial, its quantity in the finished food product, and its toxicity are all evaluated in accordance with these requirements [117]. A manufacturer’s guide titled “FDA Regulation of Nanotechnology” provides manufacturers with information on how the FDA regulates nanoparticles used in food products with reference to safety and regulatory concerns in innovative food industry technologies [118,119]. According to this guidance, nanomaterials are products having particle sizes that fall within the nanoscale range (between 1 and 100 nm) and products that have physical, chemical, or biological properties that are similar to nanomaterials [120,121]. Some of the duties fall on the manufacturers, who are in charge of keeping an eye on contaminants, physicochemical qualities, and safety. They must also submit a regulatory assessment and identify any regulatory concerns with regard to the consumption of the novel food product.

## 9. Summary and Future Trends

By-products from the food sector will open up new markets for functional food additives. One of the most significant challenges in food science and technology is the search for novel functional food components derived from natural sources. By creating valuable food by-products and boosting profitability using science and innovation, by-products of the food industry are an excellent source of proteins, minerals, fatty acids, fibre, and bioactive substances. By-products from the food industry are significant because they can be used as a key raw material for the creation of functional foods. These highly valuable compounds could be recovered from food losses and trash, but there are several issues that need to be resolved before they can be used as food additives, nutraceuticals, or dietary supplements, primarily in terms of safety and toxicity. The use of encapsulation technology is one of the current initiatives to promote ingredient stability, the controlled release of the bioactive chemicals, and an extended product shelf-life.

Depending on the nation in which they are used, the food sector must abide by various regulatory criteria that reflect its awareness of the significance of safety issues. Although there are very few negative side effects from taking natural supplements, authorities must exercise caution because goods are increasingly being tampered with to include bioactive ingredients. In addition to the absence of regulatory or legal guidelines for the utilization of food waste, this research compares the regulatory environments and outlines the risk assessment standards that bioactive chemicals associated with food must adhere to in order to maintain product safety. Consumer attitudes, perceptions, and behaviours towards food waste are evolving, and as consumers’ mindsets shift towards “natural” substances for a healthy lifestyle, industry and national authorities are under more pressure to produce goods that are safe. Utilizing food industry by-products effectively can assist in lowering costs, lessening environmental damage, and showing that the food sector is sustainable, all of which have an immediate influence on the national economy. The food sector now supports a culture and nation with zero waste.

## Figures and Tables

**Table 1:** Various microencapsulation techniques and the processes involved in each technique [23,24,25].

Technique	Major Steps in Encapsulation	Reference
Spray drying	Preparation of the dispersion Homogenization of the dispersion Atomization of the infeed dispersion	[26,27]
Spray chilling	Preparation of the dispersion Homogenization of the dispersion Atomization of the infeed dispersion	[28]
Spray cooling	Preparation of the dispersion Homogenization of the dispersion Atomization of the infeed dispersion	[28]
Extrusion	Preparation of molten coating solution Dispersion of core into molten polymer Cooling or passing of core- coat mixture through dehydration liquid	
Fluidized bed coating	Preparation of coating solution Fluidization of core particles Coating of core materials	[28]
Lyophilization	Mixing of core in coating solution Freeze-drying of the mixture	[29,30]
Coacervation	Formation of a three-immiscible chemical phases Deposition of the coating Solidification of the coatings	[24,25]
Centrifugal suspension separation	Mixing of core in a coating material To obtain encapsulated micro particles, pour the mixture over a revolving disc. Drying	[25]
Inclusion complexation	Preparation of complexes by mixing or grinding of spray- drying	[31]

**Table 2:** Development of value-added food products from different waste parts of fruits and vegetables.

Raw Materials	Waste Part	Value-Added Product	Improvements Reported in Functionality	References
Apple (*Malus pumila*)	Pomace	Gluten-free cracker Ice cream	Especially beneficial to celiac disease sufferers, these items are high in dietary fibre, antioxidants, and minerals.	[49,49]
Tamarind (*Tamarindus indica* L.)	Seed	Cookies and mango juice	Natural antioxidants improve the nutritional benefits.	[50]
Banana (*Musa sp.*)	Peel	Orange juice	Increased antioxidant activity	[51,52]
Grapes (*Vitis sp.*)	Pomace	Yogurt cheese	Antioxidant capabilities, anti-inflammatory activities, anti-cancer properties, antibacterial qualities, and cardiovascular safety properties;	[53,54]
		Bread	support for type 2 diabetes, atherosclerosis, cancer, and cardiovascular disease prevention;	[55]
		Meat	Antioxidant properties;	[56]
		Cheese	Improved nutritional properties, sensory attributes like friability and adhesiveness	[57]
		Fermented milk	Natural antioxidants	[58]
Beetroot (*Beta vulgaris* L.)	Pomace	Candy and biscuit	Rich in betalain, antioxidant, and phenolics Increased pathogen resistance, anti-inflammatory effect, and antioxidant activities	[59,60]
Raspberry (*Rubus idaeus*)	Pomace	Fruit purees	Antioxidant, antimutagenic, anticarcinogenic, antibacterial, and antiviral properties	[61]
Orange (*Citrus sinensis*)	Peel and pulp	Carrot juice	Improved functional quality and shelf life	[62]
Mango (*Mangifera indica*)	Seed Kernel	Mango powder	Natural antibiotic and antifungal properties	[63]
Pomegranate (*Punica granatum* L.)	Peel	Curd and cookies	Increase the anti-oxidative attributes and shelf life of the product Antioxidant, antimicrobial & nutraceutical properties	[64,65]
Pineapple (*Ananas comosus*)	Peel and stems	Flour	Enhance the growth of good bacteria in the human microbiota, high antioxidant activity in human gut	[66]
Tomato (*Lycopersicon esculentum*)	Peels and seeds	Butter	Extended shelf life of butter with antioxidant properties	[67]
Cauliflower (*Brassica oleracea* var. botrytis)	Leaves and stem	Apple juice beverage	Anticarcinogenic properties	[68,69]

**Table 3:** Overview of techniques for calculating bioaccessibility and bioavailability of bioactive compounds.

Analysis	Methodologies
Intestinal absorption availability	In vitro: gastrointestinal static digestion models and gastrointestinal dynamic digestion models
Pre-systemic metabolism and gastrointestinal absorption	In situ: intestinal perfusion in animals Ex vivo: gastrointestinal organs In vitro: cell culture (mainly Caco-2 cells)
Gut microbiota and the availability of intestinal absorption	In vitro: microbial fermentation models
Blood plasma bioactive chemical content	In vivo: rodents, rabbits, pigs and calves

**Table 4:** Applying encapsulation to bioactive substances recovered from agro-food wastes with the intention of reintroducing them into the food chain.

Product	Bioactive Components	Source	Encapsulation Technique	Reference
Juice and fruit salad	p-Coumaric and ferulic acids, epicatechin, and quercetin	Grape stem and leaf extracts	Microencapsulation	[107]
Yogurt	Catechin, epicatechin, quercetin, ferulic acid, gallic acid, and p-coumaric acid	Cocoa hull waste	Liposomal systems	[90,108]
Cupcakes	Betalains and polyphenols	Red pitaya peel and Pomegranate peel	Microencapsulation	[109]
Beef meatballs	Polyphenols	Pomegranate peel	Nano-encapsulation	[60]

## Data Availability

The data that support the findings of this study are available within the article.
